# Holobiont–Holobiont Interactions: Redefining Host–Parasite Interactions

**DOI:** 10.1371/journal.ppat.1004093

**Published:** 2014-07-03

**Authors:** Nolwenn Marie Dheilly

**Affiliations:** MIVEGEC (UMR CNRS/IRD/UM1/UM2 5290), Montpellier, France; The Fox Chase Cancer Center, United States of America

The term holobiont (Greek, from holos, whole; bios, life; -ont, to be; whole unit of life) describes a long-term physical association between different living organisms [1]. Theoretically, this definition encompasses all symbiotic associations (along the mutualism–parasitism continuum) spanning all taxa. However, in most cases, the term holobiont is restricted to the host and its associated mutualistic symbionts. The hologenome theory of evolution considers that the holobiont is the unit under natural selection in evolution [2], [3]. I argue that this opens new perspectives on the study of host–parasite interactions. Evidence suggests that all of the diverse microorganisms associated with the host and parasite play a part in the coevolution. This new paradigm has the potential to impact our comprehension of the development and evolution of disease.

It has been established in different model species that immune system maturation requires the presence of mutualistic bacteria [4]–[6]. The tsetse fly *Glossina moritans* carries an obligate mutualist, the bacteria *Wigglesworthia glossinidia*, which is necessary for maturation of the immune system during development [6], [7]. In vertebrates, species-specific gut bacteria are necessary for the maturation and the maintenance of a healthy immune system [4], [8]–[13]. Organisms are associated with a great variety of microorganisms, including viruses and unicellular eukaryotes, and we are starting to realize that they also play an important role in shaping a healthy immune system [14]–[16].

**Figure 1:**
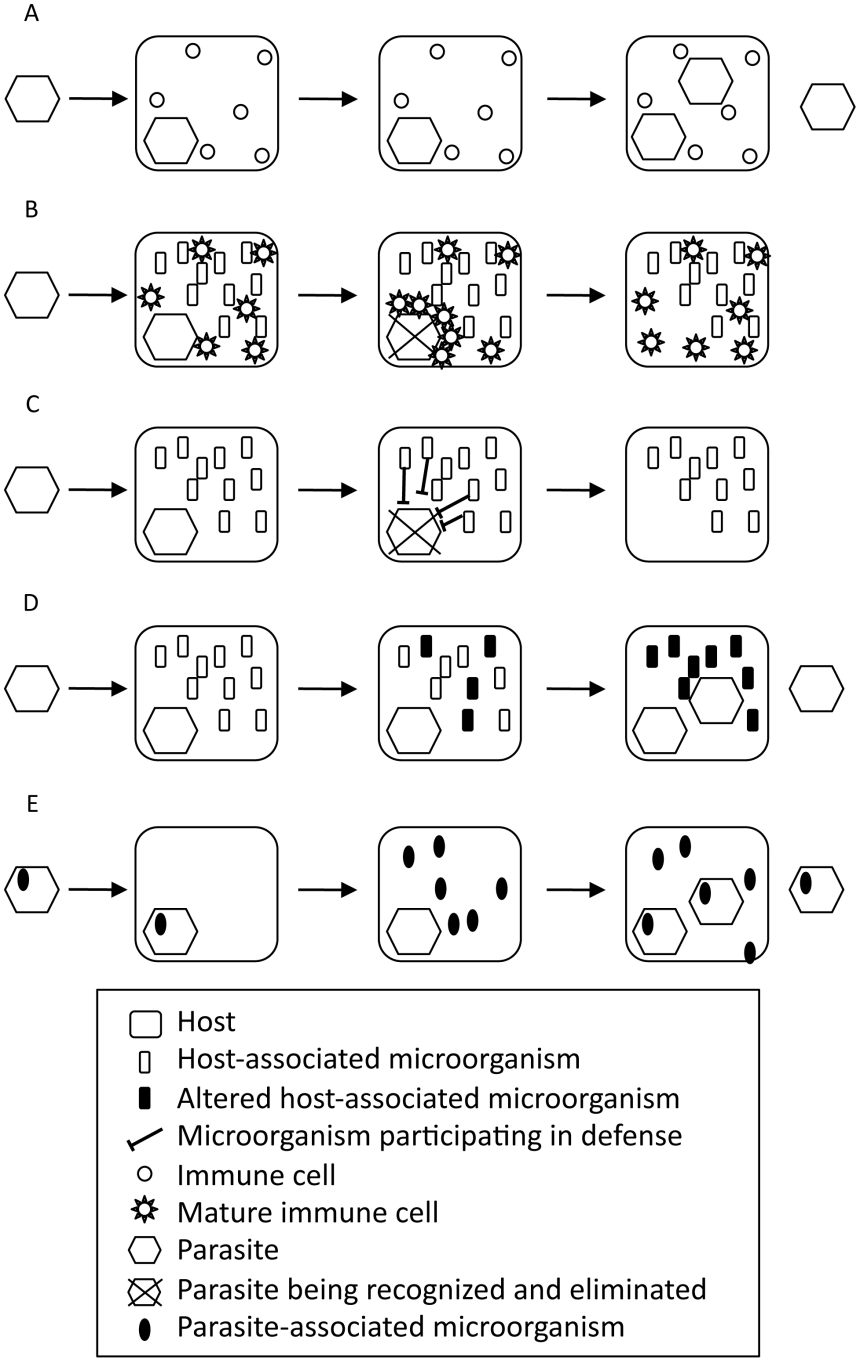
Role of microorganisms associated with the host or the parasite in the host–parasite interaction. (A) Host–parasite interaction without associated microorganisms. (B) Host-associated microorganisms participate indirectly in the immune defense by promoting immune system maturation. (C) Host-associated microorganisms participate directly in the immune defense. (D) Parasite interferes with host-associated microorganisms. (E) Parasite-associated microorganisms participate in the disease.

Thus, symbionts indirectly protect the host against various pathogens via immune activation (Figure 1A, 1B). In some cases, even parasites improve the fitness of their host; this process is called conditional mutualism [17]. For example, the hepatitis G virus limits the progression of HIV to AIDS [18], [19], the hepatitis A virus suppresses infection by the hepatitis C virus [20], and the murine cytomegalovirus protects mice against infection by *Listeria monocytgenes* and *Yersinia pestis* [21].

Host-associated microorganisms also contribute directly to the defense against pathogens (Figure 1C). The bacteriophage carried by the bacteria *Halmitonella defensa, Acyrthosiphon pisum* secondary endosymbiont (APSE) is a conditional mutualist of the pea aphid *A. pisum* [22]–[24]. It encodes toxins targeting the developing larva of the parasitic wasp *Aphidius ervi* [25], [26]. Human gut bacteria directly antagonize bacterial pathogens by producing antibacterial factors, by competing for elements necessary for pathogen growth (competitive exclusion), and by limiting their adhesion to host cells [9]. In addition, mucus-associated bacteriophages participate in the first line of defense against bacteria in various species, from cnidarians to mammals [27]. Thus, the “holo-immunome” must be studied for a comprehensive understanding of host resistance to infections.

Host-associated microorganisms are also affected by parasitosis (Figure 1D). In the coral *Oculina patagonica*, infection by *Vibrio shiloi* induces coral bleaching by directly attacking the photosynthetic microalgal endosymbionts [28], [29]. Symbiotic bacterial communities associated with the lichen *Solorina crocea* are also affected by the fungal parasite *Rhagadostoma lichenicola* [30]. HIV and SIV infections are frequently associated with gastrointestinal disorders that can be explained by an alteration of the gut microbial community [31]–[33]. As discussed above, such disruptions of host–symbiont interactions favor pathogenesis, therefore indirectly participating in the disease.

Finally, parasites are also associated with microorganisms that will directly benefit from an improved fitness of their parasitic host. These symbionts can directly participate in the disease caused by the parasite (Figure 1E). For instance, parasitoid wasps of the Ichneumonidae and Braconidae families have independently evolved mutual associations with DNA or RNA viruses (unpublished work) and play an essential role in the parasite's success and evolution [34]–[35]. Entomopathogenic nematodes are associated with bacteria that produce toxins that help degrade tissues for the nematode to feed on [36], [37]. Similarly, the plant-pathogenic fungi *Rhizopus* sp. has an endosymbiotic bacteria that produces toxins that have a key role in the disease [38].

Until recently, the role of parasite-associated microorganisms in human diseases had been underestimated, but examples are now starting to emerge. The Leishmania RNA virus promotes the persistence of *Leishmania vienna* parasites by inducing a TLR3-mediated inflammatory response that renders the host more susceptible to infection [39]. Similarly, *Trichomonasvirus*, an endosymbiotic of the protozoan parasite *Trichomonas vaginalis* is responsible for the strong proinflammatory response that causes preterm birth [40]. Microorganisms associated with such medically important parasites can now be targeted to limit the impact or development of the disease.

The theoretical framework provided by considering not only the host but also the parasite as a holobiont revealed that some interactions have been underestimated and others have not yet been explored. For example, can microorganisms associated with the host directly interact with microorganisms associated with the parasite? Can the host defend itself against infection by recognizing the microorganisms associated with the parasite? Can parasite-associated microorganisms indirectly promote the disease (by increasing its fecundity, for example)? Parasitologists, microbiologists, and immunologists have the monumental task of revealing the myriad interactions occurring between holobiont hosts and holobiont parasites. This knowledge promises to greatly impact our ability to develop new treatments and therapies.

These interactions within interactions have major implications for ecologists and evolutionary biologists, because any host–parasite interaction will be dependent on all other interactions in the system [41], [42]. The short generation time of microorganisms, along with the genetic diversity and novelty they provide [43], [44], can play an important role in the adaptation and evolution of hosts and parasites in their evolutionary arms race [45]. This coevolution may also be driven by fluctuating selection [46], in which hosts and parasites interact with different microorganisms over thousands of years, constantly evolving to favor the most advantageous symbiont at the time. In addition, associated microorganisms may be pathogenic to non-adapted individuals and drive speciation [35], [47], [48]. Thus, the study of microorganisms associated with hosts and parasites is no longer optional; it is, rather, an obligatory path that must be taken for a comprehensive understanding of the ecology and evolution of hosts and parasites. It is a necessary step for the prevention and prediction of disease outbreaks.
